# North-Eastern Hospital for Children

**Published:** 1906-12-15

**Authors:** 


### NORTH-EASTERN HOSPITAL FOR CHILDREN.

The Nurses' Home and the laundry, both of which were urgent necessities at this hospital, have at last been built, and the hospital is now able to conduct its maintenance work with increased economy. The outlay on the two buildings amounts to £11,500, of which £2,500 remains as a debt. Help is very badly needed to pay this sum and to make good a serious deficiency in income which is caused by a falling off in donations and extreme ill-fortune in the matter of legacies. This lack of recognition of the good work of this hospital is no doubt due to its situation, which is in a district with a population of half-a-million of the poor, hardworking class, and far removed from the observation of the rich. The necessary expenditure in affording relief to the poor children of the densely crowded neighbourhood is £11,000 a year, and nearly the whole of this sum has to be raised in voluntary contributions. The committee are now making strenuous efforts to raise £3,300 to make both ends meet for 1906. Secretary, Mr. T. Glenton-Kerr, Hackney Road, Bethnal Green, E.

